# Gut microbiota metabolites positively impacts chemotherapy effects in colorectal cancer

**DOI:** 10.1007/s10565-026-10147-6

**Published:** 2026-01-24

**Authors:** Sara Gomes, Sara Granja, Luana A. Osório, Ruth E. Mackay, Fátima Baltazar, Elisabete Silva, Ana Preto

**Affiliations:** 1 CBMA - Centre of Molecular and Environmental Biology, Department of Biology, University of Minho, Campus de Gualtar, 4710-054 Braga, Portugal; 2 IBS - Institute of Science and Innovation for Bio-Sustainability, University of Minho, Braga, Portugal; 3 School of Medicine, ICVS - Life and Health Sciences Research Institute, University of Minho, Braga, Portugal; 4 ICVS/3B’s - PT Government Associate Laboratory, Braga/Guimarães, Portugal; 5 Department of Life Sciences, Brunel University, London, UK; 6 REQUIMTE/LAQV, ESS, Polytechnic of Porto, Rua Dr. António Bernardino de Almeida, 4200-072 Porto, Portugal; 7 Department of Pathological, Cytological and Thanatological Anatomy, ESS|P.PORTO, 4200-072 Porto, Portugal; 8 Department of Mechanical, Aerospace and Civil Engineering, Brunel University, London, UK

**Keywords:** Short-chain fatty acids (SCFA), 5-fluorouracil (5-FU), Colorectal cancer (CRC), Chemotherapy

## Abstract

Colorectal cancer (CRC) remains one of the leading causes of cancer-related deaths worldwide, largely due to late-stage diagnosis and limited efficacy of current therapies. 5-Fluorouracil (5-FU) is the standard chemotherapeutic agent used in CRC treatment; however, its effectiveness is often hampered by resistance, toxicity, and suboptimal outcomes in advanced-stage tumors. Recent evidence suggests that gut microbiota-derived short-chain fatty acids (SCFAs) exert anticancer effects and may hold promise as therapeutic adjuvants. In this study, we investigated the potential of a physiologically relevant mixture of SCFAs to enhance the efficacy of 5-FU against CRC. Using a combination of 2D monolayer cultures, 3D models, and the in vivo chicken chorioallantoic membrane (CAM) assay, we demonstrated that SCFAs positively affect the antitumor effects of low-dose 5-FU. SCFAs contributed to the inhibition of CRC cell growth, proliferation, survival, and migration, with an overall increase of the anti-tumour effects observed across the different models. The combined treatment led to a significant reduction in tumour size in the CAM assay, contributing for an improvement of the effects of 5-FU alone. To our knowledge, this is the first report showing that physiologically relevant SCFA combinations can be harnessed to improve the therapeutic index of 5-FU in CRC, in a context-dependent manner. These findings support the development of microbiota-targeted co-adjuvant strategies to optimize CRC chemotherapy, reduce treatment toxicity, and improve patient outcomes, which is important given the clinical interest in microbiome-chemotherapy interactions.

**Graphical Abstract:**

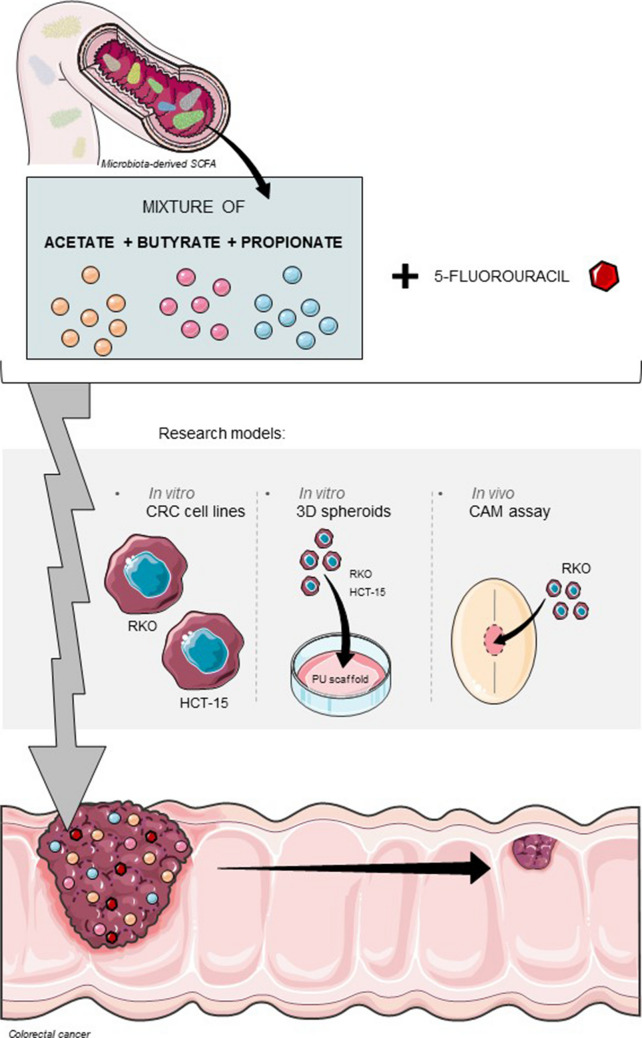

**Supplementary Information:**

The online version contains supplementary material available at 10.1007/s10565-026-10147-6.

## Introduction

The World Health Organization reported that colorectal cancer (CRC) has become one of the most common malignant tumours worldwide, responsible for approximately 1.93 million new cases and 930 600 cancer-related deaths, ranking the third in terms of incidence and the second in terms of mortality in 2020 (Sung et al. 2020; Observatory 2022). While the high incidence rates can be explained by several lifestyle factors, such as a low-fibre diet, smoking, lack of exercise and obesity, the high mortality rates are associated with the late diagnosis and therapeutic challenges, namely the resistance to chemotherapy, severe side effects and lack of effective therapies for advance-stages tumours (Sung et al. 2020; Rawla et al. 2019; Oppelt et al. 2021; Gomes et al. 2022a).

Currently, when a patient is diagnosed with CRC, there are several therapeutic options clinically available. In early stages of the disease, the first line of treatment consists in achieving the complete removal of the tumour and metastases by surgery (Xie et al. 2020). However, when the cancer is diagnosed at an advanced stage with metastases, the best option is to shrink the tumour, inhibiting the tumour spread and growth by chemotherapy or, in rectal cancers, radiotherapy (Xie et al. 2020). Common chemotherapeutic approaches include both single-agent therapy, which is mainly fluoropyrimidine fluorouracil-based, and multiple-agent regimens containing several drugs, including oxaliplatin, irinotecan, and capecitabine (Xie et al. 2020). Immunotherapy with the PD-1 inhibitors nivolumab and pembrolizumab, and targeted therapy regimens using biological agents, namely bevacizumab, panitumumab, cetuximab, ramucirumab and regorafenib are also alternatives used in the treatment of metastatic CRC. However, despite all the advances regarding the available therapeutic options, 5-fluorouracil (5-FU) remains the most used chemotherapeutic agent, both as a single strategy or in combination with other drugs, and so the need for new strategies to increase its efficacy is of extreme importance (Xie et al. 2020).

The recent recognition of the colon microbiota as a hallmark of cancer revealed the impact that these microorganisms can have in the protection against cancer development, with a clear role in malignant progression and response to therapy (Xie et al. 2020). It is already known that the adoption of specific dietary patterns, such as the ingestion of dairy products and fibres, modulates the intestinal microbiota composition towards a high percentage of short-chain fatty acids (SCFA)-producing bacteria (Observatory 2022; Rawla et al. 2019; Oppelt et al. 2021; Gomes et al. 2022a). In this regard, in the past few years, the individual role of acetate, butyrate, and propionate in the regulation of different biological processes has been investigated, in order to develop new alternatives for CRC treatment. Considering the effects against CRC cells, our group has already reported that acetate plays an anticarcinogenic role, since it is able to inhibit CRC cell proliferation and induce cell death by apoptosis, with promotion of lysosome membrane permeabilization (LMP) and release of cathepsin D (Gomes et al. 2020; Marques et al. 2013; Ferro et al. 2016; Oliveira et al. 2015). Butyrate has the ability to inhibit cell differentiation, induce cell death by apoptosis, and block cell proliferation by promoting cell-cycle arrest (Gomes et al. 2020; Li et al. 2017; Queirós et al. 2012; Donohoe et al. 2012; Zhang et al. 2010; Fung et al. 2012). Propionate suppresses CRC development by inducing anti survival mechanisms namely apoptosis, necrosis, or autophagy. In addition, this metabolite is also able to inhibit inflammatory responses and to modulate histone acetylation (Gomes et al. 2020; Tedelind et al. 2007; Hague et al. 1995). The individual role of acetate, butyrate and propionate has been widely studied during the past decade; however, the understanding of their combined effects was only recently demonstrated for the first time by our research group (Gomes et al. 2022b). The combination of three SCFA at the ratio found at human physiological conditions (60% acetate, 15% butyrate and 25% propionate) revealed to have potential therapeutic effects against CRC cells (Gomes et al. 2022b). When in mixture, SCFA exhibit an additive profile, being able to inhibit cell growth in a dose-dependent manner, with higher specificity to CRC cells rather than non-tumorigenic cells (Gomes et al. 2022b). The study reported, for the first time, that SCFA in combination impact the regulation of several biological processes (Gomes et al. 2022b), and inhibit CRC cell proliferation in a more significant way than individually.(Gomes et al. 2022b). Moreover, the combination of these metabolites is able to induce apoptotic cell death with lysosome membrane permeabilization and consequent cytosolic acidification (Gomes et al. 2022b). The reported antitumoral effects induced by the microbiota-derived SCFA suggest that they can potentially contribute to the development of new strategies for CRC therapeutics based on the increase in the SCFA levels. In this regard, the general aim of the present work is to investigate the possibility of using SCFA as co-adjuvants to 5-FU, the most commonly used chemotherapeutic agent in CRC treatment.

## Material and methods

### Cell lines and culture conditions

The work was carried out in two cell lines derived from human colorectal cancer (CRC), RKO and HCT-15 (Table 1). RKO cell line was first described by Michael Brattain and is derived from a primary colonic carcinoma (Brattain et al. 1984). These cells are microsatellite-unstable and harbour a BRAF mutation (Preto et al. 2008). They were cultured in Dulbecco’s Modified Eagle’s Medium (DMEM) (Biochrom, Berlin, Germany), supplemented with 10% (v/v) heat inactivated fetal bovine serum (FBS; Biochrom, Berlin, Germany) and a mixture of penicillin streptomycin at a final concentration of 1% (v/v) (5,000 Units/mL of penicillin and 5,000 μg/mL of streptomycin). HCT-15 cell line is derived from an adenocarcinoma specimen of human colon, removed during the normal course of a surgery (Dexter et al. 1979; Tibbetts et al. 1977). They are microsatellite-unstable and harbour a KRAS mutation (Preto et al. 2008). These cells were grown in RPMI 1640 medium (with stable glutamine) (Biochrom, Berlin, Germany) supplemented with 10% (v/v) heat inactivated fetal bovine serum (FBS; Biochrom, Berlin, Germany) and a mixture of penicillin–streptomycin at a final concentration of 1% (v/v) (5,000 Units/mL of penicillin and 5,000 μg/mL of streptomycin). All cell lines were grown and maintained in 25 cm^2^ or 75 cm^2^ tissue culture flasks at 37 °C, under a humidified atmosphere containing 5% CO_2_.

**Table 1:** Most relevant genetic phenotypes for RKO and HCT-15 cell lines (Berg et al. 2017)

Cell line	KRAS/BRAF mutation	MMR/MSI status	p53	Baseline TYMS
RKO	BRAF V600E	MSI-high/mismatch repair deficient	Mutated/non-functional TP53	High nuclear TS expression
HCT-15	KRAS G13D	MSI phenotype	Mutated p53	Low or absent nuclear TS (no nuclear TS detected)

### Short-chain fatty acids (SCFA) and 5-fluorouracil (5-FU) solutions

Sodium acetate, sodium butyrate, sodium propionate, and 5-FU powders were purchased from Sigma Aldrich (catalogues #241,245, #303410, #P1880 and #F6627). SCFA solutions were used as supplied and stock solutions made up in deionized sterile water. 5-FU stock solution was made in DMSO. Stock solutions were at least 20 times more concentrated than the highest concentration tested, in order to prevent media dilution. Subsequent dilutions were freshly prepared before each experiment. All solutions were stored at 4 °C.

### Sulforhodamine B assay

The cytotoxic effects of each SCFA were determined using the Sulforhodamine B (SRB) assay, which allows cell density determination based on the measurement of cellular protein content (Vichai and Kirtikara 2006). Cells were seeded in 48-well plates at a density of and 5.0 × 10^4^ cells per well (for RKO and HCT-15), in a volume of 300 μL of complete culture medium. After 24 h, cells were subjected to a pre-treatment with one of three different concentrations of the SCFA mixture (IC_10_, IC_25_, and IC_50_) as well as with fresh complete medium in the conditions without the SCFA mixture (Gomes et al. 2022b). 24 h later, cells were treated with fresh complete medium as negative control, as well as with the three concentrations of the SCFA mixture (IC_10_, IC_25_, and IC_50_), three concentrations of 5-FU (IC_10_, IC_25_, and IC_50_—1.0, 2.3, and 5.0 µM for RKO cell line and 5.5, 7.6, and 93.4 µM for HCT-15 cell line) or the combination of them both. Each individual plate also included three replicates of negative controls (no test agents). 48 h after the treatment, cells were washed with 1 × PBS and fixed in methanol containing 1% acetic acid (v/v) at −20 °C for, at least, 90 min. Then, the fixing solution was carefully removed, and the plates allowed to dry at room temperature (RT). When completely dried, plates were incubated with 0.5% (w/v) SRB dissolved in 1% acetic acid (v/v) at 37 °C protected from light. 90 min later, the SRB solution was removed, and the plates washed with 1% acetic acid (v/v) in order to remove the excess of SRB. Plates were then left to air dry at RT. Finally, 1 mL of 10 mM Tris pH 10 was pipetted to solubilize the SRB, plates were carefully agitated, and 200 µL of the final solution were placed in a 96-well microplate where the absorbance was read at 540 nm in a Molecular Devices SpectraMax Plus 384 Microplate Reader (Molecular Devices, San Jose, CA, USA).

Statistical analysis was performed using GraphPad Prism version 8.4.3 for Windows, GraphPad Software, La Jolla California, USA, www.graphpad.com. To reduce inter-experimental variability, data were scaled between 0% (negative controls) and 100% effect (positive controls). Results were graphically presented as percentage of inhibition of cell growth versus concentration (mM). All individual compounds were tested in at least three independent experiments, running in triplicates.

### Colony formation assay

RKO and HCT-15 cell lines were seeded in 6-well plates at a density of 500 cells/mL and 600 cells/mL, respectively. After adhering for 24 h, cells were subjected to a pre-treatment with the IC_10_ of the SCFA mixture as well as with fresh complete medium in the conditions without the SCFA mixture. 24 h later, cells were treated with fresh complete medium as negative control, as well as with the IC_10_ of the SCFA mixture, the IC_10_ of 5-FU and the combination of them both (RKO cell line: 17.0 mM of SCFA mixture, 1.0 µM of 5-FU and 17.0 mM of SCFA mixture + 1.0 µM of 5-FU; HCT-15 cell line: 2.5 mM of SCFA mixture, 5.5 µM of 5-FU and 2.5 mM of SCFA mixture + 5.5 µM of 5-FU). After 48 h of treatment, the medium was replaced with fresh medium. Cells were then allowed to grow for 5–7 days (the medium was changed every 3 days). The colonies were washed with PBS and fixed for 30 min with 6% glutaraldehyde and 0.5% crystal violet. The number of colonies was counted using ImageJ Software, and the percentage of colonies was normalized against the negative control.

### Cell cycle analysis

Cell cycle was analysed through the measurement of the DNA content. RKO and HCT-15 cell lines were seeded in 12-well plates at a final density of 5 × 10^4^ cells/mL. After adhering for 24 h, cells were subjected to a pre-treatment with the IC_10_ of the SCFA mixture as well as with fresh complete medium in the conditions without the SCFA mixture. 24 h later, cells were treated with fresh complete medium as negative control, as well as with the IC_10_ of the SCFA mixture, the IC_10_ of 5-FU and the combination of them both. After 24 h of treatment, cells were collected, resuspended in 500 µL PBS and incubated on ice for 15 min. An amount of 1.5 mL of 96% (v/v) cold ethanol was added, and the cells were incubated for 15 min on ice. Then, cells were washed, resuspended in 500 µL of PBS, and incubated with 50 µL of RNase A solution (200 µg/mL in sodium citrate 1% (w/v)) at 37 ◦C for 15 min. An amount of 50 µL propidium iodide (PI) staining solution (0.5 mg/mL in sodium citrate 1% (w/v)) was then added and the cells were vortexed and incubated at room temperature for 30 min in the dark. PI mean fluorescence was analysed by flow cytometry using PE-A channel.

### Wound healing assay

RKO and HCT-15 cell lines were seeded in 6-well plates at a density of 5 × 10^5^ cells/mL. After adhering for 24 h, cells were subjected to a pre-treatment with the IC_10_ of the SCFA mixture as well as with fresh complete medium in the conditions without the SCFA mixture. 24 h later, cells were treated with fresh complete medium as negative control, as well as with the IC_10_ of the SCFA mixture, the IC_10_ of 5-FU and the combination of them both. At this point, a confluent cell monolayer was formed, and a wound was made by manually scratching with a 10 µL pipette tip. Cells in suspension were removed, and adherent cells were washed once with PBS. The “wounded” areas were photographed at 4 distinct places, at 40 × magnification by phase contrast microscopy. The same areas were subsequently photographed to monitor wound closure after 12, 24 and 48 h. The relative migration distances were then analyzed using Image J Software. Relative wound closure was calculated for each time point. Results are presented as percentages of wound closure and represent the mean ± SD of at least 3 independent experiments.

### 3D cell culture in polyurethane scaffolds and fluorescence microscopy

Cells were cultured electrospun biocompatible polyurethane (PU) scaffolds, specifically designed to support 3D structures. Polyurethane fibrous scaffolds were used as a 3D culture system, as previously developed and characterized by Osório et al. (Osório et al. 2021, 2024). These scaffolds present a highly porous and reproducible fibrous architecture, with controlled fiber diameter and interconnected porosity that support cell adhesion, infiltration, and spheroid formation, while allowing adequate oxygen and nutrient diffusion throughout the construct. Scaffolds were produced in consistent batches and placed on round glass coverslips prior to cell seeding.

A cover slip was coated with 4 cm^2^ scaffold and inserted in each well of a 24-well plate and washed once with a 70% (v/v) ethanol solution and twice with deionised water. Then, the plates were sterilised in ultraviolet (UV) light for 15 min. The plating and treatment of the cells were performed as previously described for the 2D experiments—cells were seeded in 24-well plates at a density of and 5.0 × 10^4^ cells per well (for RKO and HCT-15), RKO cells were incubated with 17.0 mM of SCFA mixture, 1.0 µM of 5-FU or with the combination of both. HCT-15 cells were incubated with 2.5 mM of SCFA mixture, 5.5 µM of 5-FU or with the combination of both. The growth of RKO and HCT-15 cells in the scaffolds was proved by fluorescence microscopy and SRB B assay. Spheroid analysis by fluorescence microscopy was performed using a Leica DM4000 microscope, with appropriate filter cubes: DAPI (blue) and Cy3 (red) with a 40 × objective and a 100 × oil immersion objective. Images were processed using the Leica Application Suite Advanced Fluorescence (LAS F) software.

### Chicken chorioallantoic membrane (CAM) assay

CAM assay was performed as previously described (Hagedorn et al. 2005). Briefly, fertilized chicken eggs (supplied by PintoBar, Portugal) were incubated at 37 °C in a humidified atmosphere, and on day 3 of development, a window was made into the eggshell after puncturing the air chamber, and eggs were sealed with BTK tape and returned to the incubator. On day 9 of development, 2 × 10^6^ RKO cells were re-suspended on 10 µL of Matrigel (BD Biosciences, Massachusetts, USA), placed on the CAM, and the eggs were tapped and returned to the incubator. At day 13, eggs were subjected to a pre-treatment with the IC_50_ of the SCFA mixture as well as with fresh complete medium in the conditions without the SCFA mixture. At day 14, eggs were treated with fresh complete medium as negative control, as well as with the IC_50_ of the SCFA mixture, the IC_50_ of 5-FU and the combination of both. On developmental days 13, 14 and 17, tumours were photographed *in ovo* using a stereomicroscope (Olympus S2 × 16). At day 17, chicken embryos were sacrificed at − 80 °C for 10 min. CAMs and tumours were dissected, fixed in 4% paraformaldehyde at room temperature, and photographed *ex ovo*. The area of the tumours and blood vessels from a selected area containing the tumour were quantified using the Image J software. A total of 59 RKO fertilized chicken eggs were used, 16 in the control group, 15 in the group treated with the SCFA mixture only, 13 in the group treated with the 5-FU only and 15 in the group treated with the combination of the SCFA mixture with 5-FU.

### Immunohistochemistry

Histological slides with 4 μm-thick tissue sections were subjected to immunohistochemistry using a polymer system (UltraVision ONE Detection System: HRP Polymer Lab Vision Corporation, Fremont, CA, USA). Regarding the protocol, deparaffinized and rehydrated slides were incubated with 10 mM citrate buffer (pH 6.0) for 15 min in a microwave at 600 W for antigen retrieval.The sections were then incubated overnight at room temperature with a primary anti-Ki-67 antibody (Biolegend ref: 350,502, dilution 1:100). The immune reaction was visualized using 3,3′-Diamonobenzidine (DAB Substrate Kit Abcam (ab64238)) as chromogen. The stained slides were evaluated and photographed using the Olympus BX6F microscope with a 40 × immersion objective.

### Regression modelling and statistical analysis

Nonlinear regression analysis of all SCFA, individually or in mixture, was performed using a best-fit approach (Scholze et al. 2001). The data obtained from the different experiments were fitted to appropriate dosimetric models (Logit, Weibull, or General Logit I) using GraphPad Prism version 8.4.3 for Windows, GraphPad Software, La Jolla, California USA, www.graphpad.com. All of the nonlinear regression models describe sigmoidal concentration–response relationships. A suitable best-fit model was selected based on a statistical goodness-of-fit principle, after independently fitting each equation to the same data set. All data provided are from at least three independent experiments, run in triplicates. All data was normalized in relation to the vehicle control.

## Results

### Short-chain fatty acids mixture contributes the inhibition of colorectal cancer cells growth induced by 5-fluorouracil

**Fig. 1:**
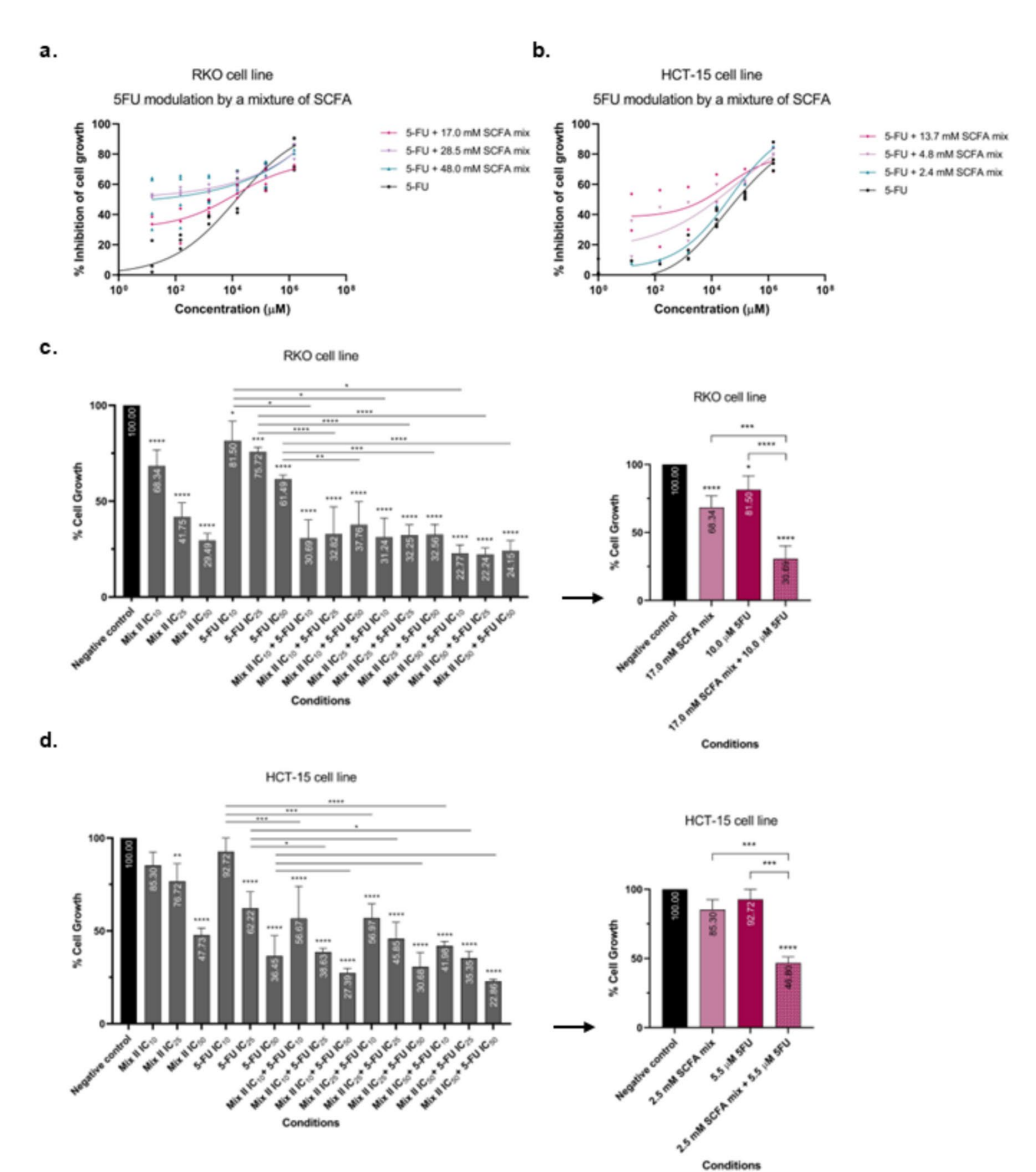
Evaluation of the effects induced by the combination of SCFA and 5-FU in the growth of RKO and HCT-15 cell lines by SRB assay. (**a**) Regression model for 5-FU cytotoxic effects in RKO cell line. The solid lines represent the regression models obtained in the SRB assay for the 5-FU alone (black line) or combined with 17.0 mM (pink line), 28.5 mM (violet line) and 48.0 mM (blue line) of the SCFA mixture following 48 h incubation in RKO cells. (**b**) Regression model for 5-FU cytotoxic effects in HCT-15 cell line. The solid lines represent the regression models obtained in the SRB assay for the 5-FU alone (black line) or combined with 2.4 mM (pink line), 4.8 mM (violet line) and 13.7 mM (blue line) of the SCFA mixture following 48 h incubation in HCT-15 cells. Experimental data were obtained from a minimum of three independent experiments run in triplicate. (**c**) RKO cells were incubated with 17.0, 28.5 or 50.0 mM of SCFA mixture, 1.0, 2.3 or 5.0 µM of 5-FU or with one of the different combinations of both. A simplified graphic showing only the treatments with the low doses of SCFA mix, 5-FU and the combination of both is presented. (**d**) HCT-15 cells were incubated with 2.5, 4.8 or 14.0 mM of SCFA mixture, 5.5, 7.6 or 93.4 µM of 5-FU or with one of the different combinations of both. A simplified graphic showing only the treatments with the low doses of SCFA mix, 5-FU and the combination of both is presented. All data herein presented are from at least three independent experiments run in triplicate. Statistical analysis was performed using One-Way ANOVA (p-value 0.0332 (*), 0.0021 (**), 0.0002 (***) and 0.001 compared with negative control cells and with the individual treatments

We used a regression model for the 5-FU cytotoxic effects, alone or combined with one of three fixed concentrations of SCFA mixture (a high, an intermediate, and a low concentration, as described in our previous work (Gomes et al. 2022b) (IC50 values described in Table 2). 5-FU concentrations were tested combined with a mixture of acetate, butyrate, and propionate. Here, the SCFA were combined according to the physiological molar ratio found in the human intestine, constituted by 60% of acetate, 15% of butyrate and 25% of propionate, as previously described (Gomes et al. 2022b). For each cell line, three different concentrations of the SCFA mixture were tested: 17.0, 28.5, and 48.0 mM for the RKO cell line and 2.4, 4.8, and 13.7 mM for the HCT-15 cell line. The results were clear in demonstrating that the combination with SCFA changed the 5-FU dose–response curve, indicating that, when in a physiological mixture, acetate, butyrate, and propionate can successfully contribute for the anti-growth effect induced by 5-FU treatment (Fig. 1a, b).

**Table 2:** IC50 values calculated for the SCFA mixture and 5-FU on RKO and HCT-15 cell lines

Cell line	IC50 values	
	SCFA mixture	5-FU
RKO	47.61 mM	4.72 µM
HCT-15	13.67 mM	93.41 µM

After proving that the SCFA mixture is able to contribute to the 5-FU anti-growth effects, we intended to understand which would be the best combination to use. With that intention, we performed more SRB assays in which we tested three concentrations of the SCFA mixture (17.0, 28.5, and 48.0 mM for RKO cell line and 2.4, 4.8, and 13.7 mM for HCT-15 cell line) and three concentrations of 5-FU (1.0, 2.3, and 5.0 µM for RKO cell line and 5.5, 7.6, and 93.4 µM for HCT-15 cell line), individually and in combination. Surprisingly, for both cell lines, the combination of the lower concentrations of SCFA and 5-FU revealed to be the most promising, when compared with the individual effects, with a percentage of inhibition of cells growth of around 30% for RKO cell line and 46% for HCT-15 (Fig. 1c, d).

### Short-chain fatty acids modulate the effects of 5-fluorouracil on colony formation in colorectal cancer cells

**Fig. 2:**
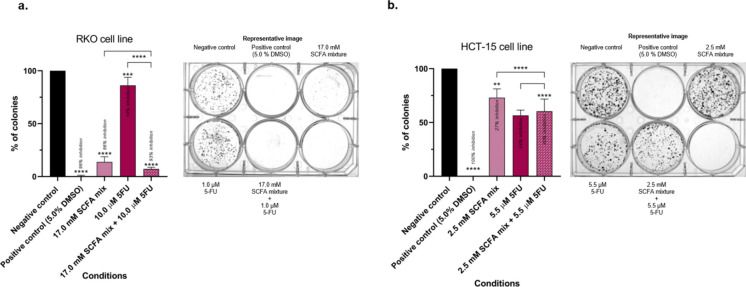
Evaluation of the effects induced by the combination of SCFA and 5-FU in the colony formation ability of RKO and HCT-15 cell lines. (**a, b**) Colony formation was measured after 5–7 days of treatment in RKO and HCT-15 cell lines, respectively. Representative images of the colonies are presented according to each condition (RKO: well a1—negative control; well a2 – positive control (5.0% DMSO); well a3—17.0 mM of SCFA mixture; well b1—1.0 µM of 5-FU; well b2—17.0 mM of SCFA mixture + 1.0 µM of 5-FU; HCT-15: well a1—negative control; well a2 – positive control (5.0% DMSO); well a3–2.5 mM of SCFA mixture; well b1–5.5 µM of 5-FU; well b2–2.5 mM of SCFA mixture + 5.5 µM of 5-FU). Statistical analysis was performed using One-Way ANOVA (p-value 0.0332 (*), 0.0021 (**), 0.0002 (***) and 0.001 compared with negative control cells or with the individual treatments

The combination of the lower concentrations of SCFA and 5-FU were tested and compared with the individual effects of each, in different phenotypic CRC hallmarks (RKO cell line: 17.0 mM of SCFA mixture, 1.0 µM of 5-FU and 17.0 mM of SCFA mixture + 1.0 µM of 5-FU; HCT-15 cell line: 2.5 mM of SCFA mixture, 5.5 µM of 5-FU and 2.5 mM of SCFA mixture + 5.5 µM of 5-FU). Firstly, the impact of the SCFA mixture on the 5-FU effects on colony formation was evaluated through a clonogenic assay. By using sub-lethal IC10 concentrations and removing the drugs before colony expansion, we minimized acute cell death during the assay. The results indicated that, in the RKO cell line, the single treatment with SCFA, per se, was able to inhibit approximately 75% of colony formation, when compared with the negative control (Fig. 2 a). When combined with 5-FU, the inhibition of colony formation was observed, although higher than SCFA alone. This seems to be due mainly to the effects of the SCFA mixture, since the individual effects of 5-FU only presented values of around 20%. Regarding the effects on the HCT-15 cell line, both the combination of SCFA with 5-FU and the individual compounds had a similar inhibitory effect against the formation of colonies (Fig. 2b).

### Short-chain fatty acids influence the cell cycle alterations induced by 5-fluorouracil

**Fig. 3:**
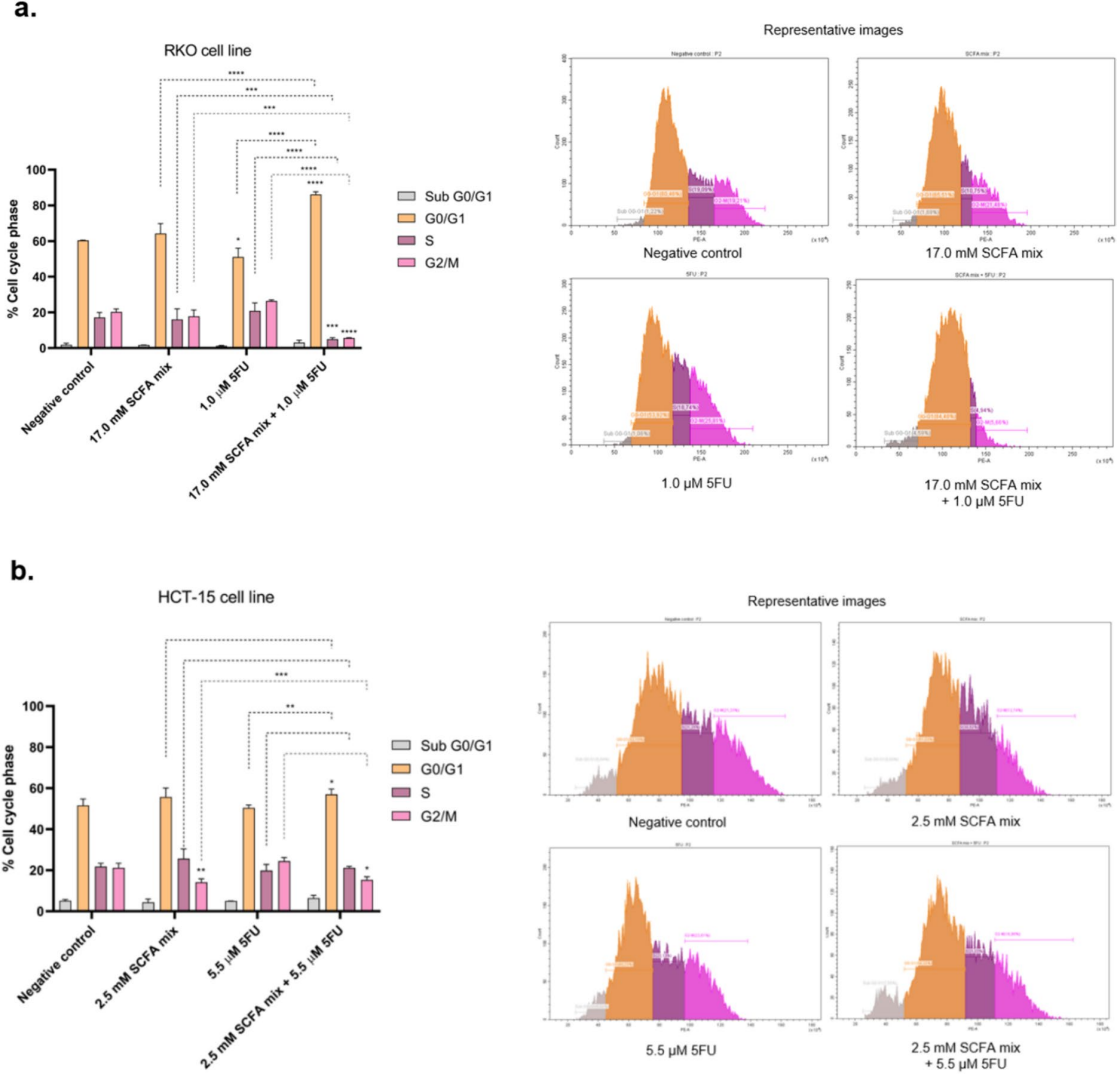
Evaluation of the cell cycle effects induced by the combination of SCFA and 5-FU in RKO and HCT-15 cell lines. (**a, b**) Cell cycle was evaluated by PI staining by flow cytometry after 24 h of treatment in RKO and HCT-15 cell lines, respectively. Representative graphs of the cell cycle analysis are presented. Statistical analysis was performed using One-Way ANOVA (p-value 0.0332 (*), 0.0021 (**), 0.0002 (***) and 0.001 compared with negative control cells or with the individual treatments

Subsequent to exploring their impact on colony formation, the impact of the SCFA mixture, individually and combined with 5-FU on cell proliferation was evaluated, through the analysis of the cell cycle by flow cytometry. For both cell lines, no significant cell death effects were induced by the treatments, as indicated by the lower percentage of the sub G0/G1 population (Fig. 3a, b). In RKO cells, it was possible to unveil that the combined treatment with SCFA and 5-FU is able to induce cell cycle arrest in G0/G1 phase, with a consequently decrease in the phases S and G2/M, when compared both with the negative control and the individual treatments (Fig. 3a). In HCT-15 cells, a similar effect was observed, however less significant (Fig. 3b). Nonetheless, phase-specific alterations were detected. The combined treatment with SCFA and 5-FU significantly increased the proportion of cells in the G0/G1 phase when compared with 5-FU alone, indicating an additional contribution from SCFA to G0/G1 arrest. Furthermore, a significant increase in the G2/M fraction was observed when compared with SCFA treatment alone, suggesting that the combination may also interfere with later stages of the cell cycle. The combined treatment induced cell cycle arrest on G0/G1 phase, that was significant, when compared with the negative control or with the effects of the individual 5-FU treatment.

### Short-chain fatty acids impact the migratory behaviour of colorectal cancer cells in the presence of 5-fluorouracil

**Fig. 4:**
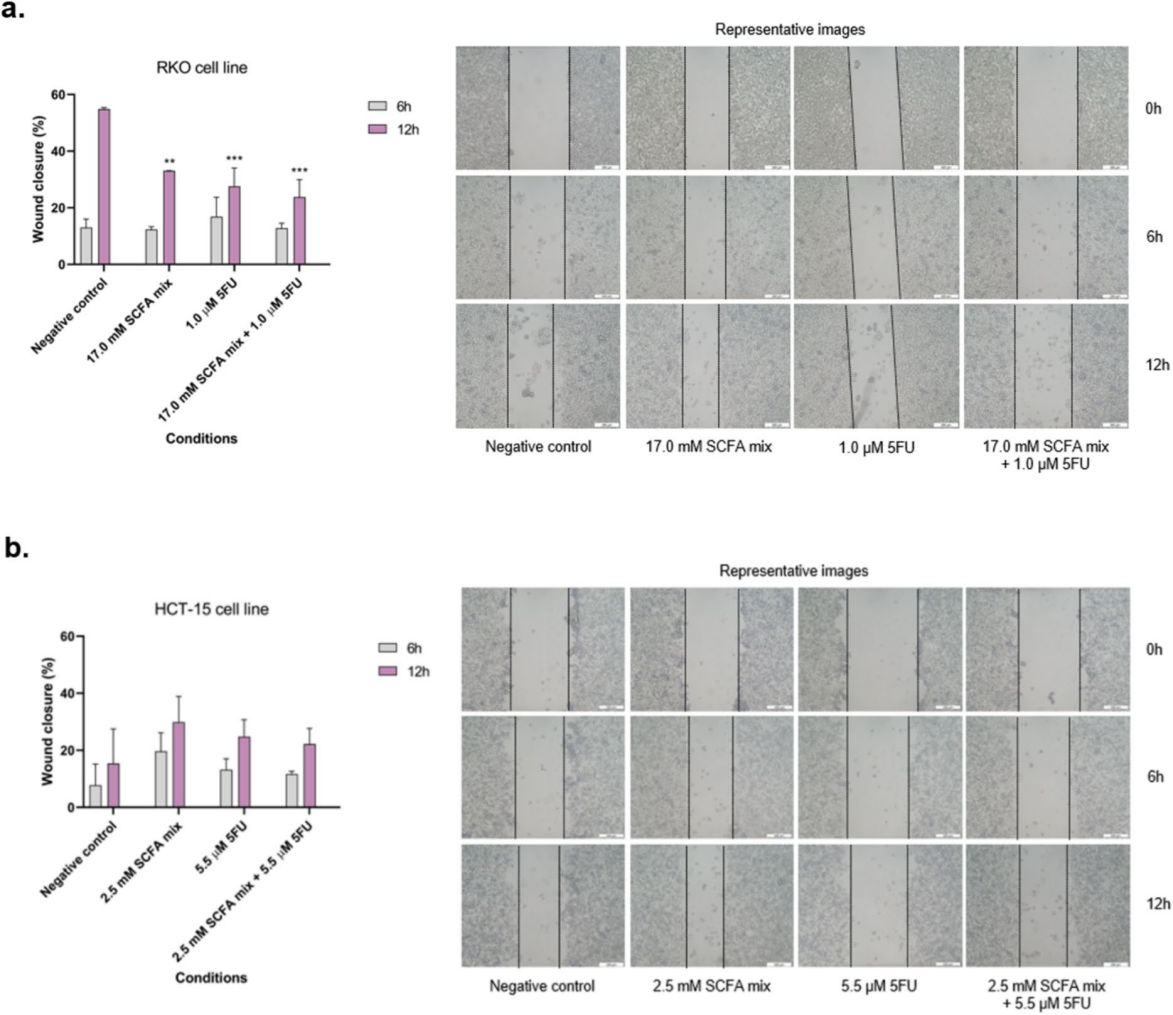
Evaluation of the effects induced by the combination of SCFA and 5-FU on the motility of RKO and HCT-15 cell lines. (**a, b**) Cell migration was assessed by scratch assay after 6 and 12 of treatment in RKO and HCT-15, respectively. Representative images of the scratch assay are presented. All data herein presented are from at least three independent experiments run in duplicate or triplicate. Statistical analysis was performed using One-Way ANOVA (p-value 0.0332 (*), 0.0021 (**), 0.0002 (***) and 0.001 compared with negative control cells or with the individual treatments

In order to understand if the combined treatment of SCFA and 5-FU have inhibitory effects on CRC cell migration, the percentage of wound closure was evaluated after 6, 12 and 24 h of treatment. For the RKO cell line, there were no significant effects induced after 6 h of incubation, however, all the treatments blocked cell migration after 12 h (Fig. 4a). In HCT-15 cells, no significant effects on cell migration were induced by SCFA, 5-FU or both at 6 h or 12 h (Fig. 4b).

### Short-chain fatty acids contribute for the effects of 5-fluorouracil using 3D *in vitro* static colorectal cancer cell culture

**Fig. 5:**
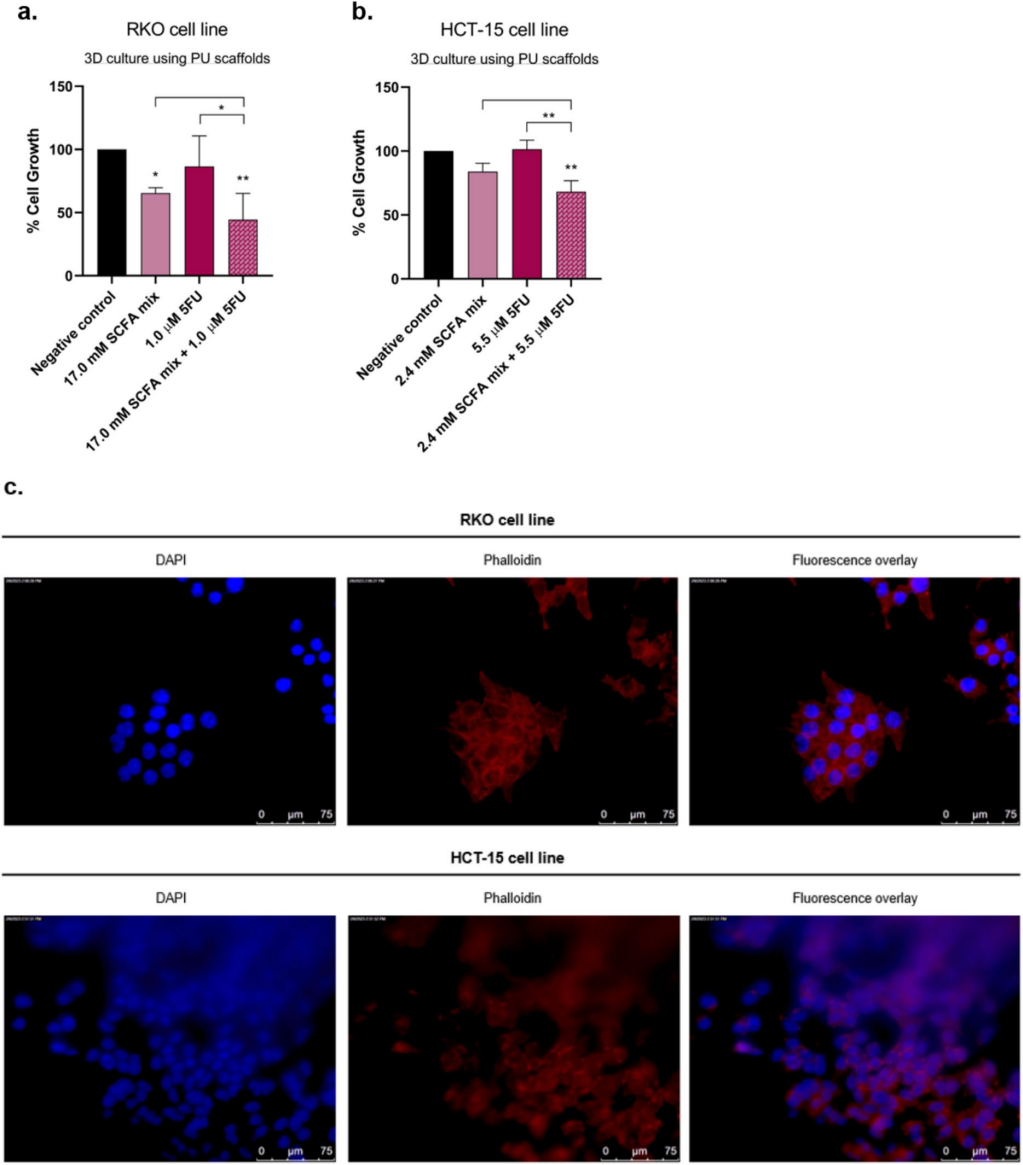
3D cultures of RKO and HCT-15 cells using PU biocompatible scaffolds. (**a, b**) Evaluation of the effects induced by the combination of SCFA and 5-FU in the growth of RKO **(a)** and HCT-15 **(b)** spheroids in PU scaffolds by SRB assay. RKO cells were incubated with 17.0 mM of SCFA mixture, 1.0 µM of 5-FU or with the combination of both. HCT-15 cells were incubated with 2.5 mM of SCFA mixture, 5.5 µM of 5-FU or with the combination of both. The data herein presented are from at least three independent experiments run in triplicate. Statistical analysis was performed using One-Way ANOVA (p-value 0.0332 (*), 0.0021 (**), 0.0002 (***) and 0.001 compared with negative control cells and with the individual treatments. **(c)** Fluorescence microscopy images of RKO and HCT-15 cells 3D-cultured in PU scaffolds after the combined treatment with 17.0 mM of SCFA mixture and 1.0 µM of 5-FU (for RKO spheroids) or with 2.4 mM of SCFA mixture and 5.5 µM of 5-FU (for HCT-15 spheroids). Cells were stained with phalloidin and co-stained with DAPI

The combined treatment with 17.0 mM of SCFA mixture and 1.0 µM of 5-FU (for RKO 3D structures) or with 2.4 mM of SCFA mixture and 5.5 µM of 5-FU (for HCT-15) revealed to be more effective in reducing the cell growth, when compared to the negative control or to the individual treatments (Fig. 5a, b). Moreover, the fluorescence microscopy analysis confirmed that both cell lines were effectively growing in the scaffolds, however, no significant morphological changes were observed between conditions (Fig. 5c).

### Short-chain fatty acids in combination with 5-fluorouracil decrease tumour growth *in vivo*

**Fig. 6:**
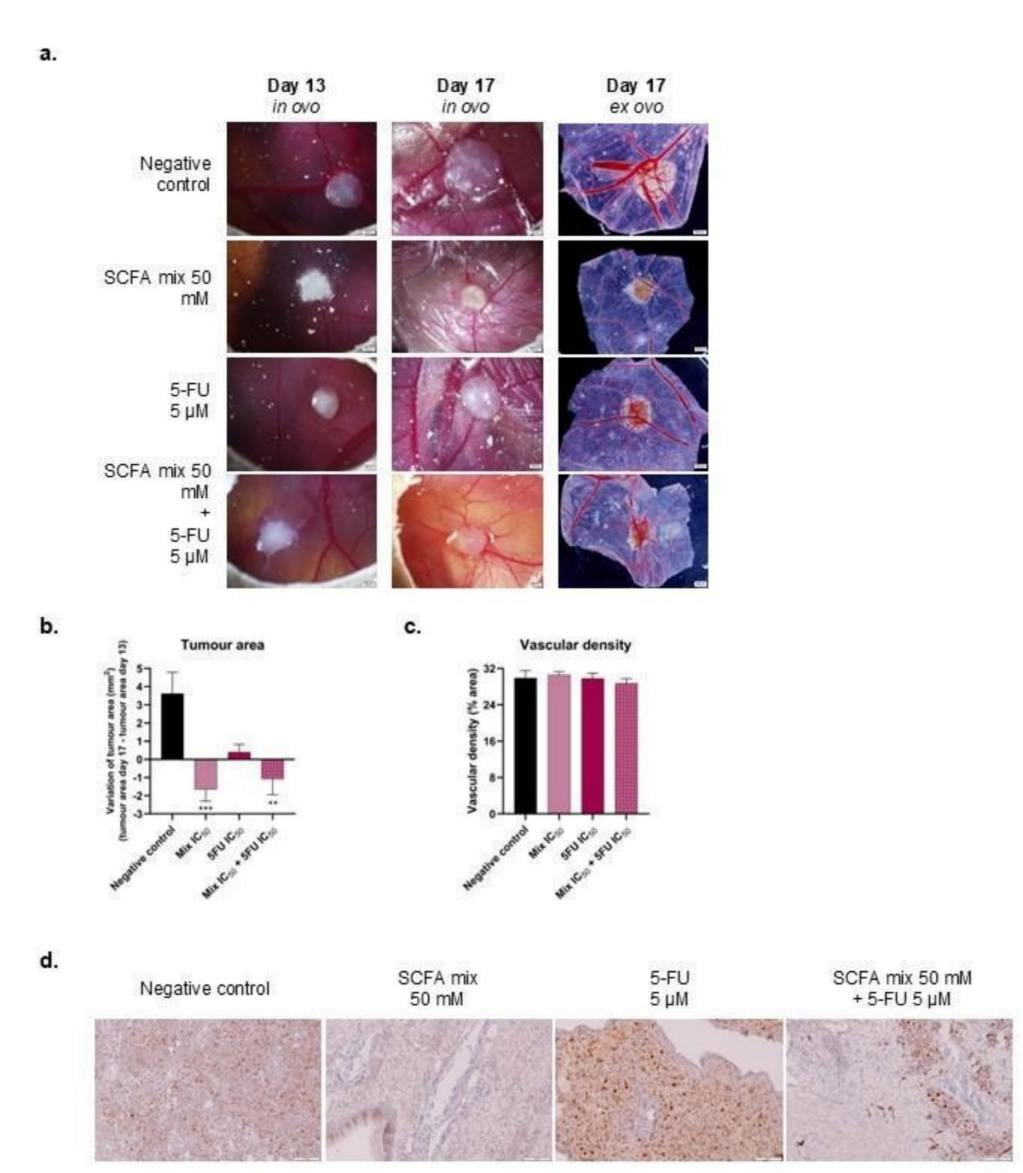
In vivo evaluation of the combined effect of SCFA and 5-FU using the CAM assay. (**a**) Representative pictures of CAM *in ovo* pre-treatments (day 13) (scale bar = 1 mm) and *in ovo* and *ex ovo* post-treatments (day 17) (scale bar = 2 mm). CAMs were pre-treated with 50.0 mM of SCFA mixture in the correspondent conditions and, 24 h later, CAMs were treated with 50.0 mM of SCFA mixture, 5.0 µM of 5-FU or the combination of both for 4 days. For the negative control group, CAMs were exposed to culture medium. (**b**) Quantification of the tumor area difference from day 13 to day 17. (**c**) Quantification of the percentage of blood vessel area. (**d**) Representative pictures of Ki-67 expression for each treated CAM condition; scale bar = 100 μm

To evaluate the *in vivo* response to the combined treatment, the chicken embryo chorioallantoic membrane (CAM) assay was used. RKO cells were implanted in the CAM of chicken embryos and allowed to grow for 4 days (from day 9 to day 13 of embryo development). On day 13, the pre-treatment with the SCFA mixture solution was administered followed by the combined treatment containing the SCFA mixture and 5-FU, 24 h later. The response to the combined treatment was compared with untreated tumours, as well as with the response to the individual treatments (SCFA alone and 5-FU alone) (Fig. 6a). As it is shown in Fig. 6(b and c), the treatment with 50.0 mM of SCFA mixture was able to decrease the tumours’ growth by about 2 mm^2^, while the single treatment with 5.0 µM of 5-FU was ineffective in decreasing the tumour growth. The combined treatment induced an approximately 2 mm^2^ average reduction in the size of RKO-derived tumours. Regarding the percentage of vascularisation around the tumours, no significant differences were observed. Additionally, we evaluated the levels of Ki-67 expression for each condition and observed that the combined treatment with both SCFA and 5-FU decreased Ki-67 expression when compared to the control group, indicating that tumor cell proliferation was inhibited (Fig. 6d).

## Discussion

Despite the recent advances in immunotherapy, chemotherapy with 5-FU remains the most commonly used approach for CRC treatment, either alone or in combination with other drugs, such as angiogenesis inhibitors (bevacizumab or ramucirumab), or epidermal growth factor receptor inhibitors (panitumumab or cetuximab) (Benson et al. 2021). However, the low survival rate, specifically for patients with a late diagnosis, lead to an urgent need for the development of new strategies to enhance the efficacy of 5-FU (McQuade et al. 2017; Okechukwu et al. 2024).

5-FU is a synthetic fluorinated pyrimidine analogue administered intravenously that requires intracellular conversion into active metabolites (Blondy et al. 2020). These active metabolites disrupt both DNA and RNA synthesis by inhibiting the action of thymidylate synthase, a critical enzyme involved in the synthesis of thymine nucleotide (Longley et al. 2003; Pardini et al. 2011). SCFA, namely acetate, butyrate, and propionate, emerged as microbiota-derived metabolites with high therapeutic potential, that specifically target CRC cells, while having low bystander effects in normal cells (Gomes et al. 2022b). Our group has already described that all three SCFA, individually or combined at the physiological proportions found in the human colon, are involved in the regulation of several biological processes in CRC cells, namely cell survival, proliferation, apoptosis, energetic metabolism, cytosolic pH and lysosome membrane permeabilization (LMP) (Gomes et al. 2022b). Given the described potential associated with the effect of SCFA on CRC cells survival, the present work was performed in order to unveil if SCFA mixture is able to increase the efficacy of 5-FU-based chemotherapy. Considering that a high percentage of patients are diagnosed with CRC containing a KRAS or a BRAF mutation (approximately 40 and 10%, respectively), all the experiments were performed in two cell lines with different genetic backgrounds, i.e., HCT-15 cell line (with a KRAS ^G12D^ mutation) and RKO cell line (with a BRAF ^V600E^ mutation) (Preto et al. 2008). At this stage, only tumorigenic cell lines were tested, since it has been already reported by our group that SCFA effects are specific towards CRC cells, as doses required for inhibiting 50% of cell growth of the cancer cell lines are lower than the doses required for NCM460 cells, a cell line with a non-tumorigenic epithelial background (Gomes et al. 2022b).

While total SCFA concentrations vary considerably along the intestinal tract and between individuals, the relative proportions among these three major SCFA remain relatively stable, and this was the rationale behind the selection of the acetate:butyrate:propionate rations to be tested (Gomes et al. 2022b). The regression model used to determine the 5-FU cytotoxic effects, alone or combined with different concentrations of SCFA, demonstrated that increasing concentrations of SCFA mixture are able to modulate the effects of 5-FU. The most promising results were obtained with the combination of the lower concentrations of both 5-FU and SCFA, indicating that there is not only a possibility to increase the efficacy of the treatment using low doses of SCFA but also that it is possible to decrease the 5-FU concentration to achieve the same anti-growth effects, suggesting the potential use of SCFA as co-adjuvants. There are already several studies reporting the synergistic effect of the combination treatment of chemotherapy and drugs in cancers. For example, combining 5-FU with kaempferol significantly suppressed colorectal cancer cell proliferation via downregulation of the PI3K/Akt pathway (Li et al. 2019 Jul), and co-treatment with 5-FU and salinomycin potentiated ferroptotic cell death in colorectal cancer by inhibiting SLC7A11/GPX4 (Wang et al. 2025).

5-FU treatment is associated with many side effects, including fatigue, loss of appetite, intestinal inflammation and diarrhea, the possibility of using lower doses can increase the efficacy of 5-FU while being beneficial to the quality of life of patients undergoing this line of treatment (Negarandeh et al. 2020).

In order to understand if this impact could be extended to a long chemotherapy regimen, the effects of the combination of SCFA with 5-FU on cell survival and proliferation were assessed through colony formation assay (Teixeira-Guedes et al. 2022). The results indicate that even after over a week after the exposure to a low dose of SCFA mixture, there was an inhibition on the formation of colonies, suggesting a promising effect in a prolonged treatment regimen, specifically in RKO cells harbouring a BRAF mutation. This difference observed after the treatment with SCFA in both cell lines could be explained based on the resistance associated with tumour cells with KRAS activating mutations, since it is already reported that a KRAS mutation status is often associated with a lack of benefit from regimens of chemotherapy, when compared with BRAF-mutated tumours (Garcia-Carbonero et al. 2020).

The inhibition of cell cycle progression in cancer cells is an important strategy in controlling cancer progression, since cancer cells are often effective in overcoming checkpoint mechanisms and, therefore, undergo a continuous proliferation cycle (Hanahan 2022; Hanahan and Weinberg 2011; Hanahan et al. 2000). Regarding the effects on cell proliferation, cell cycle analysis revealed that the combined treatment with low doses of both SCFA mixture and 5-FU blocked the cell cycle in both RKO and HCT-15 (Gomes et al. 2022b; Focaccetti et al. 2015). To the best of our knowledge, this is the first study revealing that proliferation inhibition through cell cycle arrest is affected when 5-FU and SCFA are combined at lower concentrations.

The wound healing assay was performed as a predictive method to study the inhibitory potential of the combined treatment on cell motility and migration, which is a relevant feature of cancer cell metastatic ability (Hanahan 2022; Hanahan and Weinberg 2011; Hanahan et al. 2000). The results indicated that all treatments were able to inhibit RKO cells’ motility after 12 h of exposure, revealing a potential effect in blocking the migration, and the consequent metastasis of the tumours (Jonkman et al. 2014). Regarding the invasiveness capacity of the RKO cells, proteomic characteristics of epithelial-to-mesenchymal transition (EMT), a characteristic of invasive and metastatic cancers characterized by loss of intercellular contacts, loss of basoapical polarity, gain of mesenchymal markers and increased invasive properties have previously been reported (Halvey et al. 2014). In the same study, the RKO motility/invasiveness phenotypes were evaluated and compared with another CRC-derived cell line by wound-healing and invasion assays, and the results revealed that RKO cells (BRAFV600Emutation) completely covered a scratched surface within 24 h, whereas open areas remained with SW480 cells (KRAS G12V mutation +) (Halvey et al. 2014). Previous studies have demonstrated that butyrate is able to suppress the motility of CRC cells via deactivating AKT/ERK signalling in histone deacetylase dependent manner (Li et al. 2017) and that that propionate has the potential to reduce viability and inhibit migration of glioblastoma cells (Filippone et al. 2023). It was also reported that no changes were detected in the levels of different invasiveness-related proteins, namely SNAIL, E-cadherin, and vimentin, after the exposure of COLO-205 cells to acetate (Rodríguez-Enríquez et al. 2021). However, the same cells exposed to butyrate or propionate revealed increased levels of several invasiveness proteins (Rodríguez-Enríquez et al. 2021). Regarding the potential interference of 5-FU with CRC cell motility, it is already known that this compound inhibits the migration and invasion of CRC cells, possibly through the PI3K/Akt pathway regulated by the MARCH1 protein (Wang et al. 2021). Considering this knowledge and the results previously published by our research group showing that butyrate seems to have the ability to “command” the SCFA mixture behaviour, we can hypothesize that the inhibition of CRC cells motility induced by the treatment with SCFA can be explained by the butyrate potential to block Akt/ERK signalling pathway.

The three-dimensional (3D) culture systems appeared as a complementary model to overcome some of the limitations associated with the traditional two-dimensional (2D) in vitro cell culture systems, such as the lack of cellular communication (cell–cell), interaction (cell–cell and cell–matrix) and architecture that is not representative of 3D tissues (Habanjar et al. 2021). Furthermore, in order to decrease the use of animal-derived resources we took advantage of a new biocompatible scaffold produced and optimised by our group (Osório et al. 2024). As described previously (Osório et al. 2021), even though animal-derived scaffolds are vastly used in 3D culture for their ability of replicating *in vivo* microenvironment, they are associated with a plethora of disadvantages that affect reproducibility such as unclear composition and batch-to-batch variability. Hence, given its proven low cost, stability and biocompatibility, a synthetic electrospun polyurethane (PU) scaffold was selected for this study PU is vastly known for its hydrophilicity and degradation rate which allow the scaffold to interact with cell behaviour and their microenvironment (Gao et al. 2022; Kaur et al. 2021). As shown on the fluorescence microscopy images, we were able to successfully cultivate both RKO and HCT-15 spheroids in the scaffolds with no significant alterations in terms of cell morphology. The results obtained in the 3D cell culture were able to validate the 2D cell lines results, since the SRB assay showed that the combination of low doses of SCFA and 5-FU is effective in reducing the growth of the spheroids in both cells. Interestingly, a significant decrease of cell growth was also observed in RKO spheroids treated only with the SCFA mixture, indicating that when organized in 3D, cells can be more sensitive to the effects of SCFA. In fact, this pattern was already reported in a different study, in which the exposure to the same concentrations of cetuximab in both 2D and 3D produced higher effects in the spheroids, however, the reason beyond this increased sensitivity is still unknown (Melissaridou et al. 2019).

In order to validate the use of a SCFA mixture to improve the effects of 5-FU *in vivo*, we used the well-established CAM *in vivo* model (Harper et al. 2021). CAM provides tissue responses similar to those of cell-based and animal-based assays, with the advantage that CAM is not innervated and thus no pain can be experienced by the organism (Harper et al. 2021). The usage of this animal model as pre-clinical model to study the effects of SCFA has been previously reported, where sodium butyrate-loaded nanoparticles were tested for the treatment of neovascularization in age-related macular degeneration (Reis et al. 2022). To study possible effects in CRC, RKO cells were selected for this experiment as they present the most promising results in *in vitro* assays. We were able to show that the SCFA mixture, alone or combined with 5-FU, significantly decreased the size of the RKO tumours formed in the CAM. Although the combination did not outperform SCFA alone in the CAM model, it significantly reduced tumour size compared with 5-FU alone, indicating that SCFAs may increase the effects of low-dose 5-FU even in vivo. This finding, while not demonstrating synergy over SCFAs, suggests potential clinical relevance, as enhancing 5-FU efficacy could enable dose reduction and thereby mitigate treatment-associated adverse effects.

This is a proof-of-concept that SCFA produced by the intestinal microbiota are important in the response to chemotherapy, at least to 5-FU. The concept of pharmacomicrobiomics has been investigated as a new discipline exploring the reciprocal interplay between the microbiota and drugs in the patient (Panebianco et al. 2018; Chen et al. 2022). In order to improve the therapeutic outcome and alleviate drug adverse effects, a number of approaches to selectively manipulate microbiota have been suggested, including administration of probiotics, prebiotics, synbiotics, postbiotics, and antibiotics in support of conventional treatments (Panebianco et al. 2018). Accumulating evidences have also revealed that fecal microbiota transplantation (FMT) has demonstrated remarkable clinical efficacy against several gastrointestinal diseases, namely *Clostridium difficile infection*, intractable functional constipation, inflammatory bowel diseases, and hematologic malignancies. Even though data regarding FMT in CRC patients are still limited, studies have demonstrated that the benefits of this approach include modulation of immunotherapy efficacy, amelioration of bile acid metabolism, and restoration of intestinal microbial diversity (Kaźmierczak-Siedlecka et al. 2020).

Regarding the efficacy of anticancer drugs, there are already reports showing that live wild-type bacteria can affect the efficacy of some anticancer agents either positively or negatively in vitro and in vivo, most likely via enzymatic modifications (Lehouritis et al. 2015). The cytotoxicity of several drugs, namely fludarabine and 6-mercaptopurine-2-deoxyadenosine, revealed to be increased when the bacterial concentration is higher, supporting the potential to improve therapeutic index through deliberate modification of the bacterial content of cancer patients or tumours (Lehouritis et al. 2015).

The overall composition of the microbiota, as well as substrate availability, intestinal pH, and transit time, influences the total amounts of SCFA, making microbial activity a key determinant of the SCFA pool and its physiological effects along the colon (Gomes et al. 2020). The modulation of the intestinal microbiota composition towards a SCFA-producing species enrichment can constitute a promising strategy to improve chemotherapy (Gomes et al. 2022b; Lehouritis et al. 2015). Since our results demonstrated that this increase of the 5-FU anticancer effects can be induced with low levels of SCFA, we can hypothesize that this modulation of the intestinal microbiota to produce SCFA can be done in a gentle way and with few implications in terms of side effects for patients.

Recently, our group has identified the mechanism by which SCFA are able to kill CRC cells and we concluded that the SCFA-induced apoptotic cell death is associated with the LMP and a consequent cytosolic acidification (Gomes et al. 2022b). Lysosomes are acidic organelles that participate in cellular digestion, and, in the context of cancer, they are involved in drug sequestration which lowers the cytotoxic potential of chemotherapeutics, reduces drug availability to sites of action, and contributes to cancer resistance (Halaby 2019; Sah et al. 2024). Moreover, it has been reported that the activity of multiple enzymes in lysosomes is significantly increased in many cancers when compared to normal tissues, increasing the predisposition for the acquisition of chemotherapeutic drugs resistance and thus constituting a relevant clinical problem (Tang et al. 2020). Particularly in the case of 5-FU treatment, several studies have suggested that lysosome-induced autophagy is an important resistance mechanism in different types of cancer (Sun et al. 2021; Xiao et al. 2021; Lund et al. 2017). In this regard, we hypothesise that the results herein presented showing the ability of a SCFA mixture to increase the effectiveness of the 5-FU treatment against CRC could be explained due to the induction of LMP that will decrease the resistance to 5-FU by blocking the autophagy process.

## Study limitations and directions for future work

This study represents a proof-of-concept for the potential of SCFA to enhance the efficacy of a highly used chemotherapeutic drug in CRC treatment. Along with the different indications of a synergistic effect of acetate, butyrate and propionate together with 5-FU against different CRC hallmarks, it is also important to highlight some limitations that should be taken into consideration for further studies. Although the SCFA and 5-FU combinatorial effects have been observed in several assays using different research models, this outcome was context-dependent which may require some additional mechanistic investigation. Mechanistic readouts, which may include HDAC activity, TYMS modulation, DNA-damage markers, and metabolic adaptations, can be considered in future studies. Additionally, the validation of the present findings in additional in vivo models, such as murine model, should be considered to better define the translational potential of SCFAs as co-adjuvants to 5-FU.

## Conclusions

**Fig. 7:**
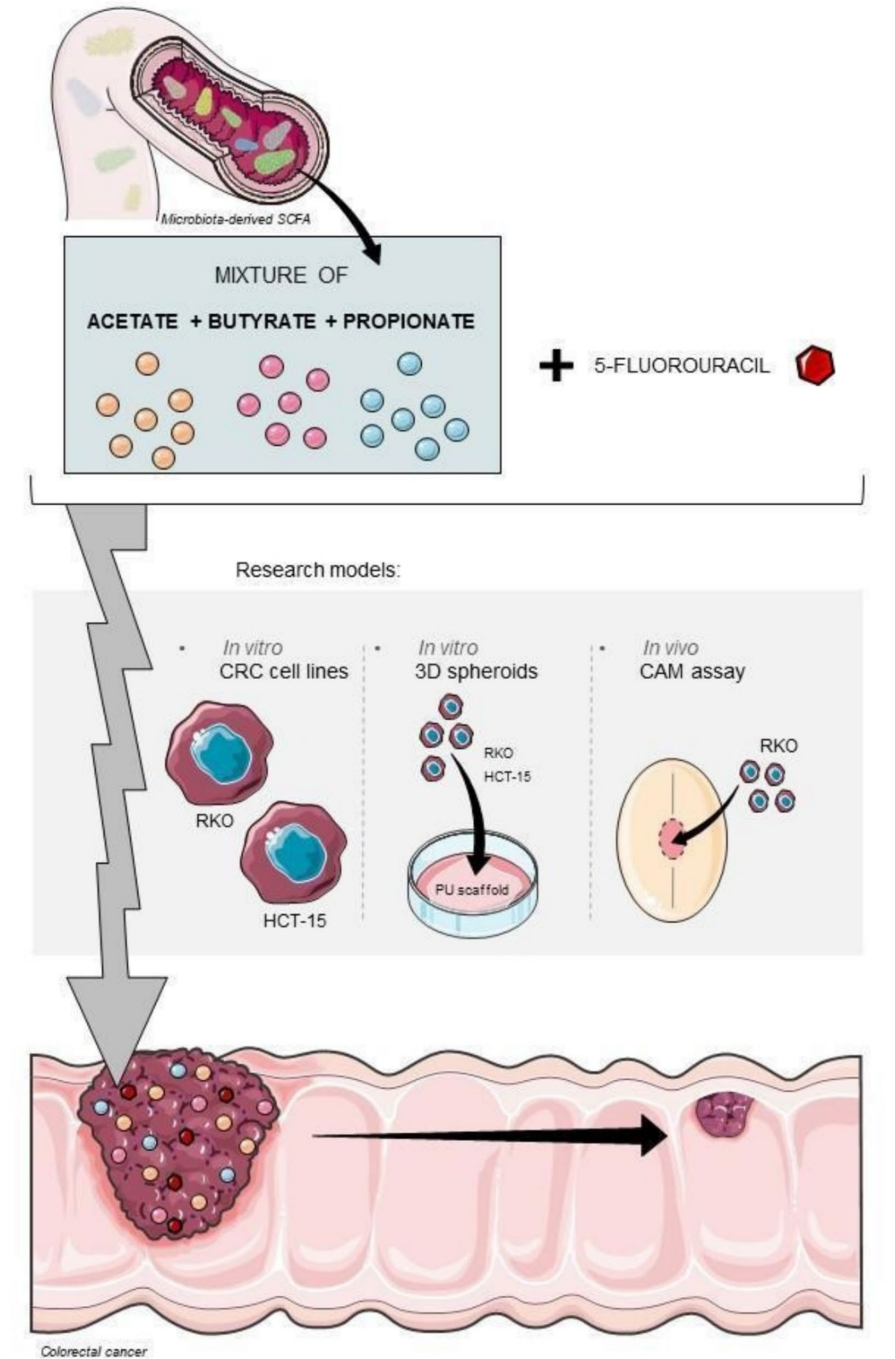
Overview of the effects of a combination of SCFA with 5-FU in CRC, using different research models

Taken together, the results herein presented suggested for the first time that microbiota-derived SCFA mixture have the potential to increase the efficacy of 5-FU, in a context-dependent manner. Importantly, this improvement is induced with low doses of both SCFA and of 5-FU, which are not harmful to normal cells, indicating that it would probably be associated with low side effects and with less resistance to 5-FU. Since our results were confirmed in different research models with different levels of complexity, we believe that there is a potential for clinical application of the combined treatment of SCFA with at least 5-FU in CRC patients (Fig. 7). We believe in the importance of promoting the increase of SCFA levels in CRC patients through the modulation of the intestinal microbiota composition towards more favourable bacteria species for SCFA production, namely using dietary supplements such as pre- or probiotics or fecal transplantation. In this way, it would be possible to improve the efficacy of the treatment of CRC patients, decreasing the concentration of the chemotherapeutic agent by the promotion of a healthier microbiota composition, with clear repercussions in terms of the success of the treatment and the quality of life of the patients.

Collectively, our results support the potential of SCFA as safe, microbiota-derived adjuvants to conventional chemotherapy, paving the way for microbiome-informed strategies to improve CRC treatment efficacy and reduce drug toxicity.

## Supplementary Information

Below is the link to the electronic supplementary material.

Supplementary file1 (PDF 15 KB)

## Data Availability

Data Availability The manuscript does not include data as electronic supplementary material.
