# Supplementary material for: Gut microbiota metabolites positively impacts chemotherapy effects in colorectal cancer
Source: Cell Biol Toxicol. 2026 Jan 24;42(1):26. doi: 10.1007/s10565-026-10147-6 (PMC12855361; doi:10.1007/s10565-026-10147-6)
Supplement: Supplementary file 1 — Supplementary file1 (PDF 15 KB) [file 10565_2026_10147_MOESM1_ESM.pdf]

Highlights

- The microbiota-derived SCFA increase the efficacy of 5-FU chemotherapy in CRC
- SCFA potentiate the effects of low doses of 5-FU in complementary research models
- Microbiota modulation can promote SCFA production thus beneficiating CRC treatment
